# Metabolic Reprogramming of Non-Hodgkin's B-Cell Lymphomas and Potential Therapeutic Strategies

**DOI:** 10.3389/fonc.2018.00556

**Published:** 2018-12-04

**Authors:** Jean-Ehrland Ricci, Johanna Chiche

**Affiliations:** INSERM U1065, C3M, Team Metabolism, Cancer and Immune Responses, Universiteé Côte d'Azur, Nice, France

**Keywords:** metabolism, Non-Hodgekins lymphoma, mTORC, mammalian target of rapamycin complex, therapeutic strategies, DLBCL–Diffuse large B cell lymphoma, folicular lymphoma, mantel cell lymphoma

## Abstract

Metabolism is a wide and general term that refers to any intracellular pathways the cell utilizes in order to satisfy its energetic demand and to support cell viability and/or division. Along with phenotypic changes, all mammalian cells including immune cells modulate their metabolic program in order to reach their effector functions. Exacerbated metabolism and metabolic flexibility are also hallmarks of tumor initiation and of tumor cell progression in a complex tumor microenvironment. Metabolic reprogramming is mainly directed by the serine/threonine kinase mTOR (mammalian target of rapamycin). mTOR exists in two structurally and functionally distinct complexes, mTORC1 and mTORC2 that coordinate environmental signals and metabolic/anabolic pathways to provide macromolecules and energy needed for survival and growth. Activation of mTORC1 is required during development, differentiation and activation of immune cells. Aberrant and persistent activation of mTORC1 is often observed in malignant B cells such as Non-Hodgkin's (NH) B-cell lymphomas. Here, we review recent insights on cell metabolism and on basic mechanisms of mTORC1 regulation and metabolic functions. We highlight the distinct mechanisms driving mTORC1 activation in the three most-common types of NH B-cell lymphomas (Diffuse Large B Cell Lymphomas, Follicular Lymphomas, and Mantle Cell Lymphomas), for which the first generation of mTORC1 inhibitors (rapalogs) have been extensively evaluated in preclinical and clinical settings. Finally, we discuss the reasons for limited clinical success of this therapy and focus on potential therapeutic strategies targeting metabolic pathways, upstream and downstream of mTORC1, that can be combined to rapalogs in order to improve patient's outcome.

## Introduction

**Figure 1:**
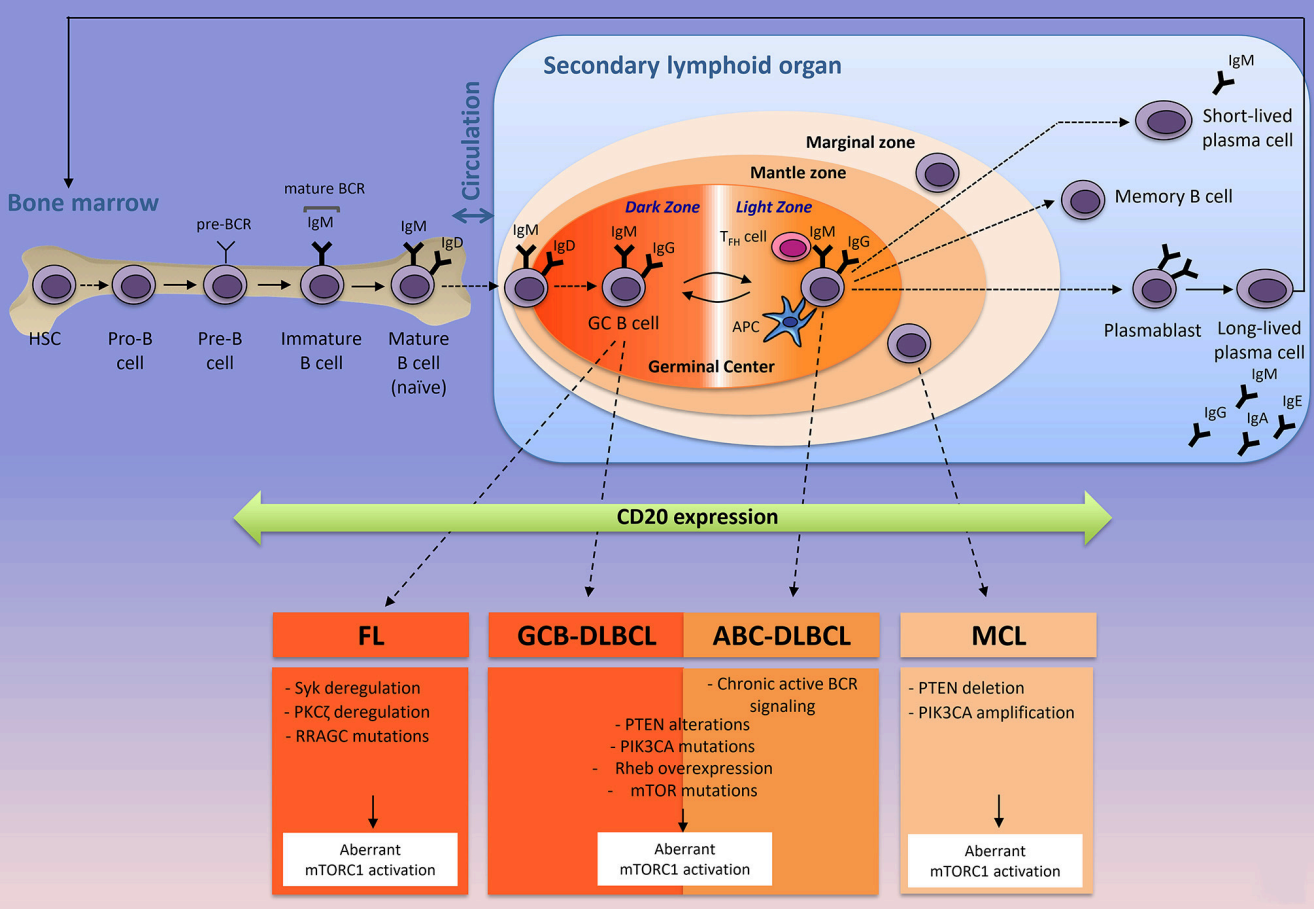
The origin of the three most-common mature B-cell lymphoid neoplasms according to their normal B cells counterparts. Naïve B cells develop in the bone marrow where they generate a B-cell receptor (BCR) and circulate to the secondary lymphoid organs (spleen or lymph nodes) where they are activated in contact with a specific antigen, resulting in a formation of a germinal center. Antibody affinity maturation occurs in the dark zone where B cells extensively proliferate and undergo somatic mutations of the immunoglobulin variable region, and in the light zones, where B cells interact with T_FH_ and APC cells and are selected for a specific clone that has the highest affinity for the antigen. MCL, DLBCL (ABC- and GCB-), and FL are NH B-cell lymphomas arise from mature B-cells in the secondary lymphoid organ. In most of the cases, FL, DLBCL, and MCL express the transmembrane protein CD20 (that is acquired from pre-B to memory stages), targeted by Rituximab (anti-CD20) and harbor different intrinsic factors leading to a constitutive mTORC1 activity. Corresponding intrinsic factors leading to aberrant mTORC1 activation are indicated. APC, antigen presenting cell; T_FH_, follicular helper T cell; Ig; immunoglobulin; BCR, B-cell receptor; FL, Follicular Lymphoma; DLBCL, Diffuse Large B Cell Lymphoma; GCB-DLBCL, germinal-center B-cell-DLBCL; ABC-DLBCL, Activated B-cell-DLBCL; MCL, Mantle Cell Lymphoma.

Immunometabolism is an emerging field of research that has already profoundly improved our understanding on how immune cells influence metabolism and *vice versa*. With the introduction and the success of immune checkpoint inhibitors in cancer treatments (anti-PD1, anti-PDL1, anti-CTLA4) [for review, see (1)], most of the scientific interest was focused on T lymphocytes due to the ability of certain T cell subsets to clear pathogens and cancer cells. In contrast, very little attention was given to decipher how the microenvironment influences metabolism of normal B cells during B cell development and how cell metabolism controls B lymphocyte fate and functions during physiological or pathological immune responses. B cells experience several phenotypic changes in order to differentiate into plasma cells and memory B cells capable of producing a large amount of antibodies (Figure 1). T and B-lymphocytes share common characteristics, as they are both able of reprogramming communications between extracellular signals, signaling pathways and metabolism, in order to reach their effectors functions. In immune cells and in cancer cells, this metabolic reprogramming is mainly regulated by the serine/threonine kinase mTOR (mammalian target of rapamycin). mTOR complex 1 (mTORC1) senses environmental changes (fluctuations of growth factors, nutrients, oxygen, immune signals) and orchestrates the cellular responses to enable cell maintenance and functions. Whereas the contribution of mTORC1 to T cell differentiation is well-established [for review, see (2)], few studies have investigated the role of mTORC1 in B cell development. In the low-oxygenated environment of the bone marrow, B cell progenitors experience a series of developmental steps to progressively express multiple immunoglobulin receptors (B-cell commitment) that will enable mature B cells to recognize a variety of foreign proteins. At early stages of their development, murine pro- and pre-B cells display a highly active mTORC1 signaling when compared to late pre-B, immature or mature B cells (3). Genetic disruption of mTORC1 activation or chemical inhibition of mTORC1 signaling leads to the accumulation of pre-B cells that are unable to produce energy levels compatible with B-cell development (3). Once mature, B cells leave the bone marrow and migrate to the secondary lymphoid organs (spleen or lymph nodes) where they can be activated upon stimulation of their B-cell receptor (BCR) by soluble (T-cell independent activation) or membrane-bound antigen (T-cell dependent activation) (Figure 1). In the context of T-cell dependent activation (leading to the strongest antibody responses), B cells bind to an antigen *via* its BCR and present antigenic peptides to T follicular helpers (T_FH_), previously stimulated by antigen presenting cells (APC) at the naïve stage. Simultaneously, B cells receive signals from T_FH_ cells through co-stimulatory molecules (such as CD40/CD40L for example) and cytokines produced by T_FH_. Once B cells are activated, they differentiate into two-ways. Activated B cells may exit the follicle, proliferate and differentiate, giving rise to short-lived plasma cells producing low-affinity antibodies (IgM or IgG) for early defense against the antigen, while long-lived plasma cells producing high-affinity antibodies are generated (Figure 1). Activated B cells proliferate and the signals provided by the crosstalk between T and B cells, help for the development (and the longevity) of germinal centers, where B cells express BCR with different antigen affinities (through somatic hypermutation and class switch recombination) and are selected for antibodies with the greater antigen affinity (antibody affinity maturation step). Antibody affinity maturation is a dynamic process occurring in two distinct zones of the germinal center. In the dark zone, germinal center B (GCB) cells express BCR with different affinities for the antigen and extensively proliferate. Antigen-dependent signals are delivered in the light zone, where B cells compete with each other for antigen, in contact with APC and T_FH_ cells (Figure 1). The cycling of B cells between the light zone and the dark zone, leads to a positive selection of a specific B cell clone harboring a BCR capable of binding the antigen with high affinity. During affinity maturation, mTORC1 activity is required *in vivo* to induce the anabolic program that enables the activated B cells, proliferation in the dark zone, but it is dispensable when cells have already engaged in cell division (4). Selected B cells leave the germinal centers as high-affinity long-lived plasma cells, which secrete a large amount of clone-specific antibodies, or as memory B cells (Figure 1).

Non-Hodgkin's (NH) B-cell lymphomas represent 90% of NH lymphomas (5) and originate from different stages of B-lymphocyte development and maturation (Figure 1). They consist in a heterogeneous group of diseases that differ at the genetic, histologic and clinical levels. Following the diagnosis, patients with NH B-cell lymphomas are primarily treated with a monoclonal anti-CD20 (R, Rituximab) combined with standard chemotherapy approaches (such as CHOP, Cyclophosphamide, Hydroxydaunorubicin, Oncovin, Prednisone). Rituximab is a monoclonal antibody targeting CD20, a plasma membrane phosphoprotein exclusively expressed by B-lymphocytes (normal and malignant) from pre-B cells to memory B cells (Figure 1). Upon binding to its target, anti-CD20 leads to B cell depletion through three main mechanisms: (i) inhibition of intracellular signaling pathways and induction of apoptosis, (ii) activation of the complement, resulting in a complement-dependent cytotoxicity (CDC) and/or (iii) recognition of anti-CD20 targeted B cells by immune effector cells (mostly natural killers or macrophages), thus inducing antibody-dependent mediated cytotoxicity (ADCC) (6). R-CHOP treatment has significantly improved patients' outcome, however, a significant proportion of patients with aggressive lymphomas such as Mantle Cell Lymphomas (MCL) or Diffuse Large B Cell Lymphomas (DLBCL) are refractory or become resistant to this treatment. Furthermore, indolent Follicular Lymphomas (FL) might experience disease transformation into an aggressive form of NH B-cell lymphoma and remain impossible to treat once the disease has relapsed. The medical need for new therapeutic options targeting refractory/relapsed NH B-cell lymphomas, remains unmet.

NH B-cell lymphomas display deregulated mTORC1 activity. In this review we describe the evidence for highly active mTORC1 signaling in the three most-common types of NH B-cell lymphomas: DLBCL, FL, and MCL (Figure 1). Inhibitors of mTORC1 activity (rapalogs) were extensively evaluated in refractory/relapsed DLBCL, FL and MCL and were less successful in the clinical studies than expected from preclinical studies. Rapalogs only gained approval in MCL for which all conventional chemotherapeutic strategies have failed. We highlight therapeutic strategies that can be combined with rapalogs in order to improve the therapeutic benefit for patients with refractory/relapsed NH B-cell lymphomas.

## Overview on Mammalian Cell Metabolism and Its Regulation by mTORC1 Signaling

### Basis on Energetic Metabolism

**Figure 2:**
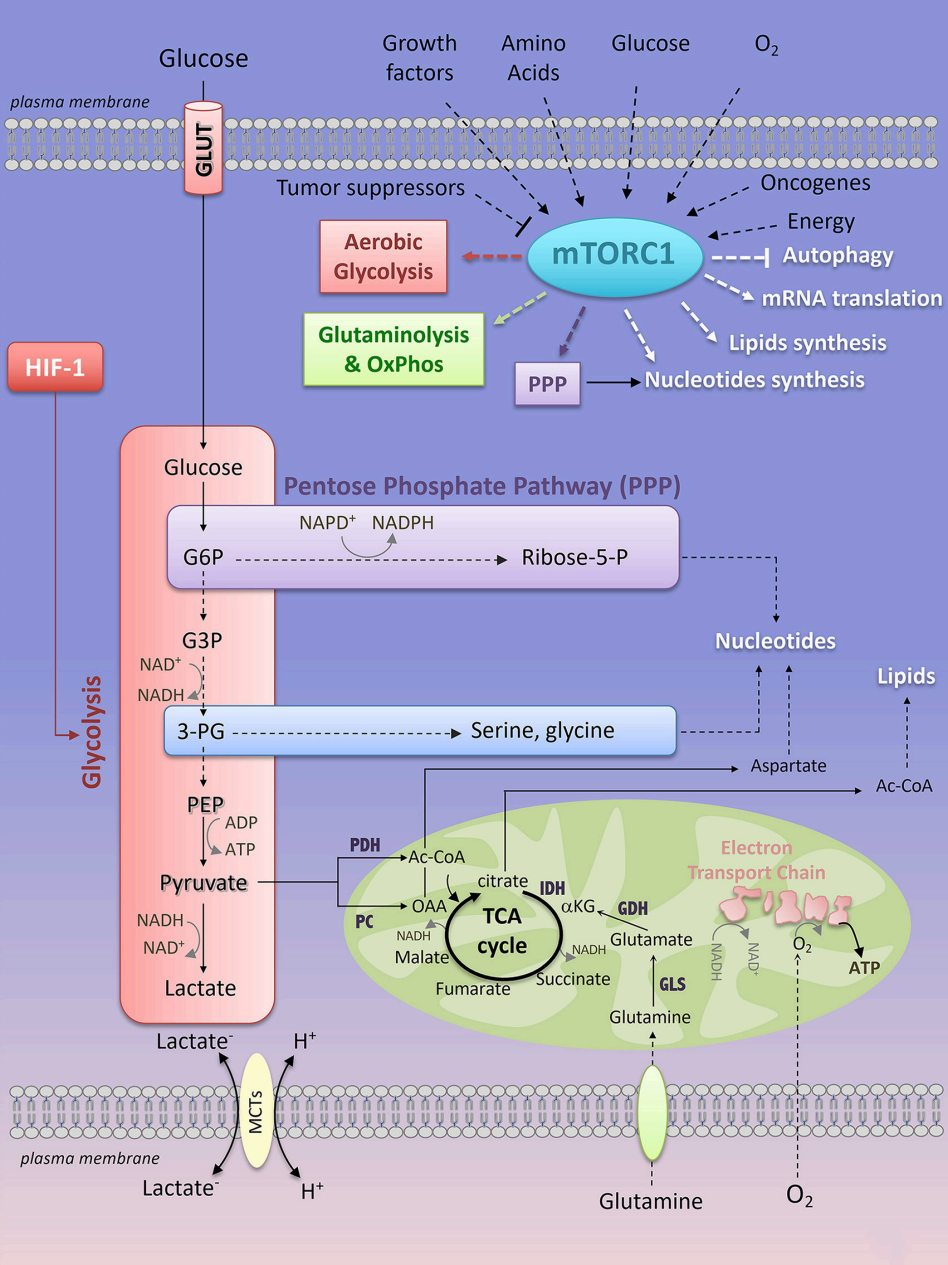
Key interconnected metabolic pathways and their relevance to mTORC1 activation. Extrinsic (growth factors, amino acids, oxygen, glucose) and intrinsic (oncogene, energy levels) factors activate mTORC1 signaling, while tumor suppressors prevent its activation. In turn, mTORC1 enhances glycolysis, through HIF-1-dependent glycolytic program, the pentose phosphate pathway, and glutamine metabolism. mTORC1-dependent regulation of cell metabolism converges through an anabolic program resulting in increased nucleotides, protein and lipid synthesis while inhibiting autophagy. mTORC1, mTOR complex 1; PPP, pentose phosphate pathway; HIF-1, Hypoxia-Inducible Factor-1; LDH, lactate dehydrogenase; PDH, pyruvate dehydrogenase; PC, pyruvate carboxylase; IDH, isocitrate dehydrogenase; GDH, glutamate dehydrogenase; GLS, glutaminase; Ac-CoA, Acetyl-CoA; OAA, oxaloacetate; α-KG, α-ketoglutarate; G6P, glucose-6-phosphate; G3P, glyceraldehyde-3-phosphate; 3-PG, 3-phosphoglycerate; PEP, phosphoenolpyruvate; Ribose-5-P, Ribose-5-phosphate; ADP, adenosine diphosphate; ATP, adenosine 5′-triphosphate; NAD^+^, nicotinamide adenine dinucleotide; NADH, reduced form of NAD^+^; NADP^+^, nicotinamide adenine dinucleotide phosphate; NADPH, reduced form of NADP^+^.

All mammalian cells (quiescent or proliferating, normal or cancerous) consume available nutrients at different rates in order to generate metabolic precursors essential for the biosynthesis of proteins, lipids, nucleotides and energy (ATP, adenosine triphosphate), thus enabling cell survival/proliferation and functions. Two main metabolic pathways, in two distinct cellular compartments, produce ATP: the glycolysis acts in the cytoplasm and the oxidative phosphorylation (OxPhos), in the mitochondria. Glycolysis converts glucose into pyruvate through a series of 10 step-reactions that transfer phosphate groups from glycolytic intermediates to adenosine diphosphate (ADP) to generate ATP (Figure 2). The pyruvate derived from glycolysis is then converted into lactate (end production of glycolysis) by the lactate dehydrogenase-A (LDH-A), which recycles the oxidized form of nicotinamide adenine dinucleotide (NAD+) in order to provide a continuous glycolytic flux (Figure 2). The bi-directional extrusion of lactic acid (H+, lactate–) outside the cells is also required to sustain a high rate of glycolysis (such as in muscle cells under exercise or in cancer cells) and is facilitated by monocarboxylate transporters (MCTs) (7) (Figure 2). Glycolysis yield 2 ATP for one molecule of glucose consumed.

The OxPhos metabolism is the route by which the tricarboxylic acid (TCA) cycle and the Electron Transport Chain (ETC) activity are connected to couple oxygen consumption to ATP production in the mitochondria (Figure 2). Mammalian cells can integrate glycolysis and OxPhos metabolism by oxidizing glucose. The pyruvate derived from glycolysis can be redirected to the mitochondria where it is converted into Acetyl-CoA (Ac-CoA) by the pyruvate dehydrogenase complex (PDH). Ac-CoA enters the TCA cycle, which generates reducing equivalents that are transferred to the mitochondrial ETC complexes as NADH (at ECT complex I) or reduced flavins (at complex II and III), thus promoting electron transport across the ECT complexes, consumption of O_2_ (complex IV) and production of ATP (complex V) (Figure 2). For 1 molecule of glucose oxidized, OxPhos metabolism consumes 6 molecules of oxygen and yields 38 ATP. Not only glucose, but also fatty acids (through fatty acid oxidation) or glutamine (through glutaminolysis) can be metabolized to provide precursors for OxPhos metabolism. Oxidation of fatty acids generates Ac-CoA that enters the TCA cycle, leading to TCA anaplerosis and mitochondrial respiration (8). Glutamine is a non-essential amino acid (NEAA) uptaken by a large family of amino acid transporters (9). Once imported into the cell, carbon-derived glutamine serves as a fuel for the TCA cycle anaplerosis. Indeed, glutamine is hydrolyzed into glutamate and inorganic ammonia *via* the activity of the glutaminase (GLS) (Figure 2). Glutamate is either extruded outside the cells against the cysteine, through the xCT antiporter (10) or is converted into α-ketoglutarate (α-KG) by the glutamate dehydrogenase (GDH), the activity of which is dependent on the essential amino acid (EAA) leucine. αKG is metabolized in the TCA cycle, thereby supporting OxPhos metabolism (Figure 2). Glutamine is also a nitrogen donor for nucleotide synthesis. The importance of glutamine in an *in vitro* culture of immune cells and of cancer cell lines derived from various tissues has been extensively reported, thus revealing glutamine as the second most consumed nutrient after glucose. However, *in vivo*, what glutamine provides to immune cells or to cancer cells remains highly debated and only partially understood. Whereas cultured lung cancer cells consume and metabolize glutamine to support OxPhos metabolism, *in vivo* glutamine only marginally contributes to TCA cycle anaplerosis and to tumor growth in mice models of lung carcinoma (11) and in patient's lung cancers (12). As a consequence, inhibition of glutaminolysis *in vivo* (using the GLS inhibitor CB-839, currently under clinical investigation in the treatment of hematologic malignancies and solid cancers including lung cancers) does not affect tumor cell progression (11). Instead, lung cancer cells preferentially use glucose-derived carbons to replenish the TCA cycle (11, 12). Glutamine-derived carbons are not prevalent to TCA cycle anaplerosis in patient-derived glioblastoma xenograft models and in glioblastoma patients (13, 14). Additionally, external sources of glutamine are dispensable for Ras-mutated tumors that rather adopted another route of nutrient uptake, a catabolic process named macropinocytosis by which extracellular proteins are internalized into the cells *via* macrovesicles. Once internalized, the macrovesicles fuse with the lysosomes and the degraded content is a “ready-to-use” amino acid supply (including glutamine) driving tumor metabolism and growth *in vitro* and *in vivo*, in mouse models of KRAS^G12D^-driven pancreatic cancers (15, 16). Macropinocytosis was recently observed in human pancreatic cancer tissues (16, 17). So far, evidence of tumor glutamine addiction was shown by PET imaging of the glutamine analog 4-^18^F-(2S,4R)-fluoroglutamine (^18^F-FGln) in preclinical models of glioma and in patients with gliomas (18). Additionally, glutamine metabolism was shown to be essential for NH B-cell lymphomas (human Burkitt cell lines) proliferation under conditions of glucose and oxygen deprivation (19).

Besides glucose, fatty acids and glutamine, other fuels such as lactate, acetate, the NEAA aspartate, asparagine and the EAA leucine, arginine and serine support cell growth in nutrient-limited environments [for details please refer to (20)].

### Metabolic Plasticity of Cancer Cells

Most mammalian cells adapt their metabolism to face unusual environmental conditions. The metabolic flexibility of a cell is a reversible phenomenon that corresponds to a “jump” from a resting metabolic state to the best-adapted metabolic pathway to sustain cellular functions in stressful environments. This metabolic choice has considerable consequences on interconnected metabolic processes and anabolic pathways. Glucose oxidation provides major carbon sources for biosynthesis. Early in the glycolysis pathway, the glycolytic intermediate metabolite glucose-6-phosphate (G6P) connects glycolysis to the non-oxidative branch of the pentose phosphate pathway (PPP), resulting in the production of ribose-5-phosphate required for *de novo* nucleotides (purine and pyrimidine) synthesis (Figure 2). Through its oxidative branch, PPP also generates NADPH that is essential for the maintenance of the cellular redox status. 3-phosphoglycerate (3-PG) is another branching point that links glycolysis to the synthesis of the NEAA serine and glycine, thereby providing one-carbon source for purines biosynthesis (Figure 2). Glucose-derived pyruvate can be converted into Ac-CoA through a reaction catalyzed by the pyruvate dehydrogenase (PDH), or into oxaloacetate (OAA) by the pyruvate carboxylase (PC) and the glutamate and oxaloacetate transaminases (GOT). Ac-CoA contributes to TCA cycle anaplerosis and is also a precursor for fatty acid and cholesterol synthesis, while OAA is a branching point from TCA cycle anaplerosis to the synthesis of NEAA aspartate and asparagine, both of which are required for protein and nucleotide synthesis (Figure 2).

What factors influence the metabolic flexibility? Like wind for bungee jumpers, metabolic requirements of mammalian cells are mainly challenged by fluctuations of nutrients (glucose, EAA, NEAA, and oxygen), growth factors and inflammatory signals in the tissue microenvironment, a fragile ecosystem in which cells from different origins (immune cells, tissue resident cells) compete for substrates (21).

In high altitudes, the metabolism of the whole body adapts to the limitation of oxygen pressure (hypoxia) in the blood (22). In a pathological context, the rapid and persistent expansion of the tumor cells led to an insufficient supply of oxygen in tumor areas that are poorly and inefficiently perfused. In these hypoxic areas, tumor cells restrict ATP production through oxidative metabolism and adapt to this energetic challenge by stabilizing the α subunit of the hypoxia-inducible factor 1 (HIF-1α), thus increasing the HIF-1-dependent glycolytic program to compensate for the lack of ATP produced by the mitochondria (23). Nevertheless, well-oxygenated cells may also use glycolysis (aerobic glycolysis). This phenomenon has been widely described as a feature of cancer cells, and is best known as the “Warburg effect.” Investigations by the Nobel laureate Otto Warburg described that cancer cells produce a larger amount of lactic acid than normal cells, even in the presence of oxygen. He concluded that oxygen is not necessarily required for cancer progression and he interpreted that cancer cells shift their metabolism to aerobic glycolysis because mitochondrial respiration is impaired (24). However, numerous studies did not support this hypothesis as they failed to demonstrate mitochondrial dysfunction as a common feature of cancer cells (25). Consequently, in most cancer cells, aerobic glycolysis is not the consequence of defective respiration. Intriguingly, why would cancer cells adopt aerobic glycolysis while it is an inefficient pathway to produce the ATP? Glycolysis allows fast ATP production and a threshold level of ATP is critical for cell survival (25). Moreover, as mentioned above, glycolysis generates a carbon source for *de novo* nucleotide synthesis, enabling rapid proliferation (26). During adaptive immune responses, metabolic flexibility is required for T cells to reach their effector functions (27, 28). Metabolic characterization of the different T cell populations revealed the metabolic flexibility of T cells with relevance to aerobic glycolysis for some T cell subsets. T helper-17 (Th17) cells engage aerobic glycolysis *in vitro* to enable their differentiation (29). In contrast, resting memory and regulatory (Treg) T cells predominantly use OxPhos metabolism (30). Proliferating T cells rely on aerobic glycolysis when activated *in vitro* (27). Thus, the Warburg effect is not a unique feature of transformed cells and is a mere reflection of cell proliferation influenced by *in vitro* cell culture conditions.

Cell-cell competition for available substrates is a dynamic process that influences the metabolism of cells. Despite a rapid access to nutrients, oxygenated tumor cells make glucose available for hypoxic glucose-addicted tumor cells to support cancer progression. As a consequence, oxygenated cells recapture hypoxic cells waste (lactate) to use it as a carbon source to supply their own energetic demand (31). Similarly, tumor cells hijack glucose from surrounding immune cells, thus restricting T cell glucose utilization, T cell metabolism and anti-cancer immune functions (21). Glycolytic tumor cells also block T effector cell functions by extruding high quantities of lactic acid, thereby contributing to tumor expansion (32).

### Same Tumor Entities, Distinct Metabolic Requirements: Identification of the Tumor Metabolic Heterogeneity

Several decades of research were dedicated to the understanding of molecular mechanisms regulating the Warburg effect in cancer cells. Oncogenes (c-MYC), loss of tumor suppressors (PTEN, p53) and activating mutations in signaling pathways, regulate glucose uptake and expression of glycolytic enzymes, thus controlling the metabolic switch toward aerobic glycolysis, as observed by Warburg (33).

Although Warburg failed to demonstrate that mitochondrial energetic functions were defective in cancer cells, mutations in the mitochondrial enzymes were later reported in a subset of tumors. As an example, mutations in the TCA cycle enzyme isocitrate dehydrogenase-1 or 2 (IDH1 or IDH2) lead to the accumulation of the oncometabolite 2-hydroxyglutarate (2-HG), responsible for aerobic stabilization of HIF-1α and subsequent increase in aerobic glycolysis (34). However, genetic drivers of aerobic glycolysis are not a feature of all cancers and some tumors subsets seem not to follow the Warburg scheme. Oxidative metabolism does contribute to tumorigenesis and to cancer progression (35), thus challenging the “Warburg concept.” With the development of “omics” technologies, such as metabolomics and fluxomics (the latter consisting in the tracing of a specific isotope to determine the rate of metabolic reactions) several studies established that aerobic glycolysis is not prevalent in tumors when grown in mice or in humans, instead *in vivo* tumor cells oxidize glucose (36) or lactate (37) to replenish the TCA cycle. Other omics approaches have provided accurate information on gene expression (gene expression profiling, GEP), thereby revealing tumor molecular and metabolic heterogeneity within the same tumor entity. The metabolic heterogeneity was first described in 2005 by a whole-genome array and multiple clustering methods, in diffuse large B cell lymphomas (DLBCL) from patients (38) and it was later confirmed in human DLBCL cell lines by a proteomic approach (39). About 30% of primary DLBCL harbor a signature of genes involved in mitochondrial metabolism (38). *In vitro*, human OxPhos-DLBCL cell lines rely on palmitate-dependent mitochondrial metabolism and are sensitive to the inhibition of mitochondrial β-oxidation (39). Since then, growing evidence of tumors relying on OxPhos metabolism to support survival and proliferation were shown in different types of cancers that originate from different tissues such as melanomas (40) pancreatic cancer (41) and lung cancer (42). Moreover, the transcription factor PGC-1α (PPARγ-coactivator-1α) involved in mitochondrial biogenesis is a marker of the metabolic state of human melanomas (40).

Such metabolic heterogeneity raises the question whether markers of the metabolic state—that remains to be found—might be clinically helpful in order to exploit the tumor metabolic vulnerabilities with relevant anti-metabolic therapies.

### Regulation of Cell Metabolism and Cell Division: Focus on mTORC1 Signaling

The metabolic program of a cell is a tightly controlled cellular process. The mechanistic target of rapamycin (mTOR), a non-typical serine/threonine kinase of the PI3K (phosphoinositide 3-kinase)-related kinase (PIKK) family, coordinates cell growth and metabolism by sensing growth factors, ATP levels and fluctuations of nutrients, in normal and in transformed cells (Figure 2). When cells encounter environmental changes incompatible with cell division, mTOR activity is shut down to reduce ATP-requiring anabolic processes (protein, nucleotides, and lipids synthesis), until the signals are newly relayed.

#### Basic Mechanisms of mTORC1 Activation

mTOR is active in two structurally and functionally distinct multi-protein complexes (mTORC1 and mTORC2) that are highly regulated. The core component of mTORC1 consists in its catalytic subunit mTOR, Raptor (a regulatory protein associated with mTOR, required for correct subcellular localization of mTORC1), mLST8 (mammalian lethal with Sec13 protein 8, involved in the stability of the catalytic domain of mTORC1) and two inhibitory subunits PRAS40 (proline-rich Akt substrate of 40 kDa) and DEPTOR (DEP domain containing interacting protein). mTORC2 also contains mTOR, mLST8 and DEPTOR but differs from mTORC1 by Rictor (rapamycin-insensitive companion of mTOR) and two regulatory subunits mSin1 and Protor 1/2. The activity of these complexes is driven by the phosphorylation of mTOR at S2448. mTORC1 and mTORC2 are activated by different signals and phosphorylate distinct substrates [for review, see (43)].

**Figure 3:**
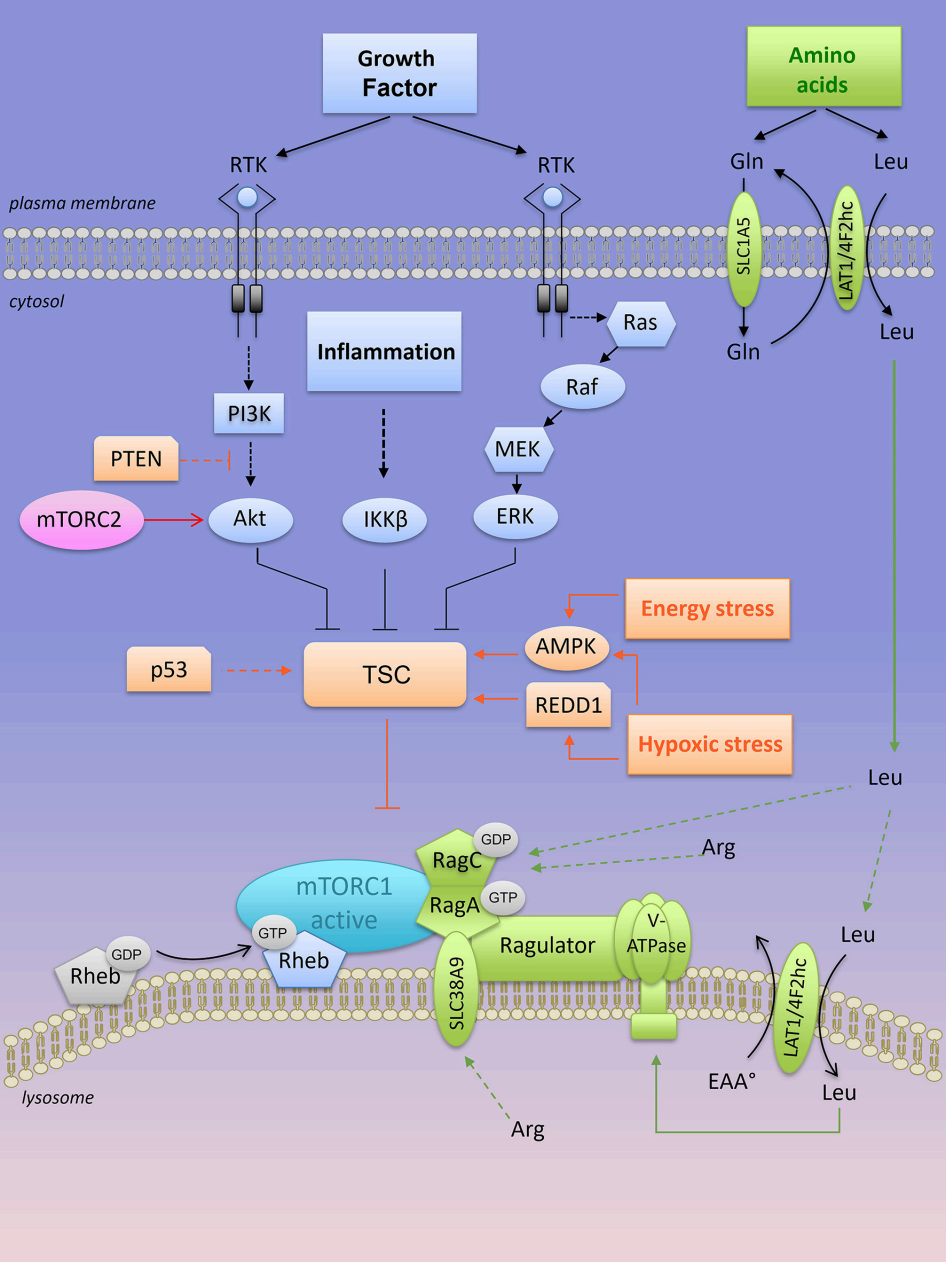
Lysosomal “inside-out” activation of mTORC1. Both growth factors and amino acids are required for a full activation of mTORC1 at the lysosome. Positive regulators of mTORC1 are shown in blue (when activated by growth factors) and in green when activated by amino acids). RTK, receptor tyrosine kinase; mTORC1, mTOR complex 1; mTORC2, mTOR complex 2; TSC, tuberous sclerosis complex; PTEN, Phosphatase and TENsin homolog; AMPK, AMP-activated protein kinase; REDD1, Regulated in DNA damage and development 1; Rheb-GTPase, Ras homology enriched in brain-GTPase; Rag, Ras-related GTP-binding protein; LAT1, L-type amino acid transporter 1; 4F2hc, CD98 heavy subunit protein; Gln, glutamine; Leu, leucine; Arg, arginine.

**Figure 4:**
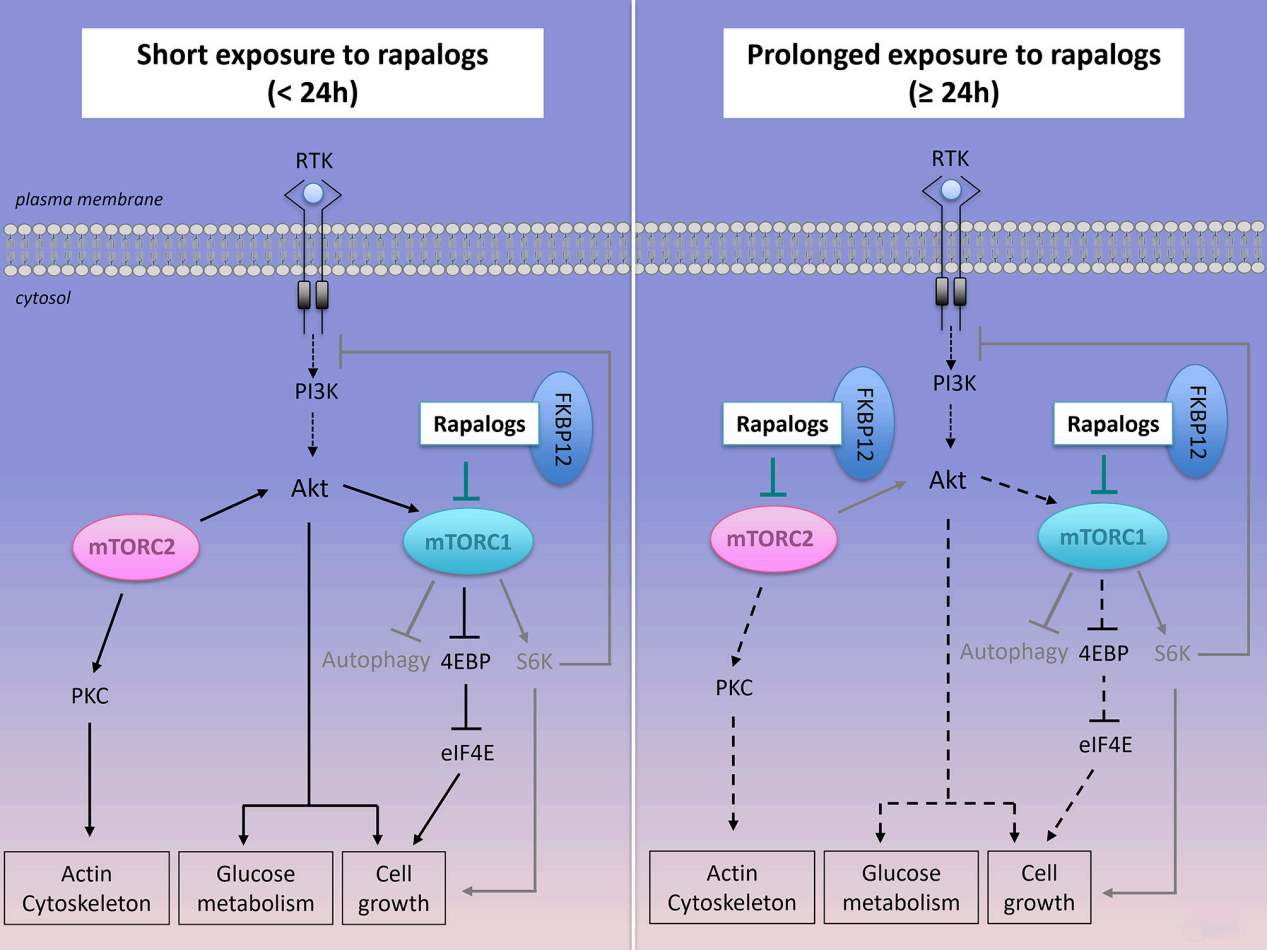
Effect of short or prolonged exposure of rapalogs on mTORC1, mTORC2 activities and on cancer survival and proliferation. Rapalogs form a complex with FKBP12 that inhibits mTORC1 activity, S6K activation but only partially reduces 4EBP signaling. Rapalogs also prevent the feedback inhibition on the PI3K/Akt signaling, thus converging toward Akt activation and cancer cell survival. Prolonged exposure to rapalogs partially inhibits mTORC2 assembly and mTORC2 functions on Akt signaling.

While little is understood concerning mTORC2 activation (44), the first two mTORC2 substrates identified so far are PKCα (Protein Kinase C α), a regulator of actin cytoskeleton (45, 46) and Akt (also known as Protein Kinase B, PKB), a serine/threonine kinase involved in cell survival and metabolism (47) (Figures 3, 4).

mTORC1 senses several independent stimuli: growth factors, intracellular ATP levels and extracellular/intracellular nutrients (Figure 3). Briefly, growth factor signals are transmitted from growth factor receptors to the PI3K/Akt pathway. Activated Akt directly phosphorylates the tuberous sclerosis complex (TSC) protein TSC2 on multiple sites, an action that inactivates the heterotrimeric TSC (which consists in TSC1, TSC2, and TBC1D7 protein) a critical regulator of the small G protein Rheb (Ras homology enriched in brain) GTPase. Both TSC and Rheb are co-localized at the surface of the lysosomes. Inactivation of the TSC complex by Akt results in a release of TSC from Rheb, which becomes Rheb-GTP and activates mTORC1 at the surface of the lysosome (Figure 3). The inflammation-activated kinase, the NF-κB regulator IκBα kinase (IKKβ) or other growth factor-activated kinases (Ras/Raf/MEK/ERK) also converge on TSC complex inhibition and mTORC1 activation through phosphorylation of TSC1 or TSC2, respectively (48–50) (Figure 3). In contrast, energy stress or hypoxia turn-off mTORC1 activity by activating TSC through induction of the metabolic regulator AMP-activated protein kinase (AMPK) and/or REDD1 (Regulated in DNA damage and development 1) (51, 52) (Figure 3). In parallel, AMPK also inhibits mTORC1 through phosphorylation of Raptor (53).

Other extrinsic factors including extracellular sources of glucose stimulate mTORC1 activity in human cancer cell lines cultivated in the presence of growth factors. Under low glucose conditions (relative to cell culture), the glycolytic enzyme GAPDH (glyceraldeyde-3-phosphate dehydrogenase) is no longer engaged in glycolysis and as a “free” enzyme it sequesters Rheb away from mTORC1 (54). Nevertheless, in the absence of amino acids, factors such as growth factors, glucose, and energy levels are not sufficient to activate mTORC1. Upon amino acid stimulation, members of the Ras-GTPase super family (Rag) are converted into their active nucleotide-bound state, resulting in a heterodimer of GTP-bound RagA or B with GDP-bound RagC or D that interacts with the multi-protein Ragulator complex (55). Rag-GTPase-Ragulator complex is tethered at the lysosomal surface and serves as a docking site for mTORC1 (Figure 3). Localization of mTORC1 at the surface of the lysosome, close to its coactivator Rheb, is required for mTORC1 activation. Thus, growth factors, in concert with amino acids, regulate mTORC1 activity. Among all amino acids, cytosolic leucine and arginine are sensed upstream of mTORC1 by Sestrin2 and CASTOR1 (Cellular Arginine Sensor for mTORC1), respectively (56–58), and they are dominantly required to support mTORC1 activation outside the lysosome (59, 60). In the presence of leucine, glutamine can also activate mTORC1 through glutaminolysis and stimulation of GTP loading of RagB (61). Later, glutamine was found to activate mTORC1 in a Rag GTPase independent manner (62). Overall, intracellular glutamine is exchanged against extracellular leucine through the plasma membrane LAT1-4F2hc transporter, thus indirectly contributing to mTORC1 activation by leucine (63). This process might explain the addiction of tumor cells to glutamine, at least when maintained *in vitro*.

A second layer of regulation resides inside the lysosome. Arginine is transported into the lysosome by the low affinity amino acid transporter SLC38A9, a transmembrane lysosomal protein that interacts with key components of the lysosomal multi complex machinery, including V-ATPases, Ragulator, Rags, and mTORC1. SLC38A9 expression is required for lysosomal arginine to activate mTORC1 (64). In a similar manner, the plasma membrane—associated leucine transporter LAT1-4F2hc (SLC7A5-SLC3A2) is recruited at the lysosomal surface to uptake leucine into the lysosomes, resulting in mTORC1 activation via lysosomal leucine-dependent stimulation of v-ATPase (65) (Figure 3).

Increased activation of mTORC1 is observed in numerous cancers due to alterations in intrinsic factors upstream of mTORC1 such as loss of function mutations in tumor suppressors (PTEN, Phosphatase and TENsin homolog, TP53, TSC1/2) or gain-of-function mutations in oncogenes (PI3K, Akt, Ras). All those alterations converge to the inactivation of TSC and constitutive active mTORC1 signaling (Figure 3). Genetic and epigenetic alterations driving aberrant mTORC1 activity in the three most common types of NH B-cell lymphomas is greater detailed in the next section (see also Figure 1).

#### The Metabolic Functions of mTORC1

Upon its activation, mTORC1 phosphorylates numerous downstream effectors, which regulate anabolic processes (nucleotide, protein and lipids synthesi, ribosome biogenesis), while suppressing catabolism (autophagy) (Figure 2). The two best-characterized substrates of mTORC1 are the ribosomal p70S6 serine/threonine kinase (S6K1) and the eukaryotic initiation factor (EIF)-4E binding protein (4E-BP1). Respectively, phosphorylation of S6K1 and 4E-BP1 increases mRNA translation initiation and cap-dependent mRNA translation, two extensively reviewed processes impacting on protein synthesis [for more details please refer to (66)]. mTORC1 can also contribute to protein synthesis by modulating the level of amino acids and/or expression of enzymes of the amino acid metabolism. For instance, asparagine sustains protein translation and enables cell proliferation in glutamine-restricted conditions (67). The levels of asparagine synthetase (ASNS) expression and of intracellular asparagine are reduced upon mTORC1 inhibition with rapamycin (68, 69).

Proliferating cells require *de novo* synthesis of nucleotides to enable DNA replication and ribosome biogenesis (where the majority of nucleotides reside). The two families of nucleotides, purine, and pyrimidine, are building blocks for DNA and RNA synthesis. mTORC1 stimulates *de novo* pyrimidine synthesis through S6K1 phosphorylation of CAD (carbamoyl-phosphate synthetase) (70), while it regulates *de novo* synthesis through ATF4 (Activating Transcription Factor 4)-dependent expression of the mitochondrial enzyme MTHFD2 (Methylene Tetrahydrofolate Dehydrogenase 2) (71). Both purine and pyrimidine synthesis require glucose-derived ribose-5-P that is generated by the PPP (Figure 2).

To meet the anabolic demand of proliferating cells (in physiological or pathological conditions), mTORC1 increases the rate of several metabolic pathways including glycolysis, the oxidative arm of the pentose phosphate pathway, lipid synthesis (72) and mitochondrial metabolism (73, 74) (Figure 2). mTORC1-dependent increase in cap-dependent translation of HIF-1α mRNA is sufficient to stimulate HIF-1-dependent glycolytic reprogramming in aerobic conditions, thus positioning the mTORC1/HIF-1 axis as a central mechanism of the Warburg effect. mTORC1 activates the oxidative arm of the PPP by regulating expression of genes involved in this metabolic pathway. *De novo* synthetized lipids are required for formation of new membranes. In a S6K-dependent or independent manner, mTORC1 activates SREBP1 (Sterol Regulatory Element-Binding Protein-1), a transcription factor regulating genes encoding for lipogenic enzymes of fatty acid and sterol biosynthesis (72, 75). Other effectors of mTORC1 contribute to oxidative metabolism. In skeletal muscle cells, inhibition of mTORC1 reduces he mitochondrial membrane potential, oxygen consumption and mitochondrial ATP as a consequence of a decrease in the genes involved in PGC-1α-regulated mitochondrial biogenesis (73). In acute T cell leukemias, mTORC1 has immediate functions on mitochondrial respiration and this regulation requires mTORC1-dependent phosphorylation of the anti-apoptotic protein Bcl-xL at the outer membrane of the mitochondria (69). In addition, mTORC1 stimulates mitochondrial biogenesis and mitochondrial energetic functions, by regulating 4E-BP-dependent translation, in a breast cancer cell line (74). Furthermore, mTORC1 promotes glutaminolysis and glutamine anaplerosis by increasing (i) GLS expression through S6K1-dependent control of c-Myc translation (76), and (ii) GDH activity, through repression of the mitochondrial Sirtuin SIRT4 that inhibits GDH (77).

Collectively, studies on mTORC1 regulations and functions in normal and in malignant cells highlight a regulatory feedback loop between mTORC1 and metabolic pathways. Consequently, drugs that would target mTORC1 and/or cancer cell metabolism are expected to impair tumor progression and to significantly improve patient survival.

## Targeting mTORC1 Signaling in Non-Hodgkin's B-Cell Lymphomas: Rationale and Clinical Results

Direct and indirect evidence of active mTORC1 signaling have been reported in several types of NH B-cell lymphomas and correlated with tumor cell proliferation and resistance to immuno-chemotherapy, thus providing a rationale for testing mTOR-targeted therapies in refractory/relapsed NH B-cell lymphomas. Here, we focus on tumor intrinsic factors leading to aberrant mTORC1 signaling in the three most common types of NH B-cell lymphomas, the high-grade MCL and DLBCL and the low-grade FL, and describe preclinical and clinical studies evaluating rapalogs (mTOR inhibitors) in the treatment of refactory/relapsed MCL, DLBCL and FL (Table 1).

**Table 1:** Summary results of phase II and III studies evaluating the effect of rapalogs as a single agent in the treatment of refractory/relapsed NH B-cell lymphomas.

**Disease**	**Drug name**	**Trial phase**	**Nbe of patients in the cohort**	**Dose administrated**	**ORR (%)**	**CR (%)**	**DR (months)**	**References**	**Year of publication**
MCL	Temsirolimus	II	35	250 mg weekly	38	3	6.9	(78)	2005
MCL	Temsirolimus	II	29	25 mg weekly	41	3.7	6	(79)	2008
MCL	Temsirolimus	III	162	175 mg weekly (for 3 weeks) followed by 75 or 25 mg weekly	22 (175/75 mg) 6 (175/25 mg) 2 (IC)	2 (175/75 mg) 0 (175/25 mg) 2 (IC)	7.1 (175/75 mg) 3.6 (175/25 mg) NA (IC)	(80)	2009
MCL	Everolimus	II	19	10 mg daily	32	10.5	NA	(81)	2011
MCL	Everolimus	II	35	10 mg daily	20	6	5.5	(82)	2012
DLBCL	Temsirolimus	II	32	25 mg	28	12.5	7.2	(83)	2010
DLBCL	Everolimus	II	47	10 mg daily	30	0	NA	(81)	2011
FL	Temsirolimus	II	39	25 mg	54	25	12.5	(83)	2010
FL	Everolimus	II	8	10 mg daily	38	12.5	NA	(81)	2011

*ORR, overall response rate; CR, complete response; DR, duration response; MCL, Mantle Cell Lymphoma; DLBCL, Diffuse Large B Cell Lymphoma; FL, Follicular Lymphoma; IC, investigator's choice; NA, not available*.

### Aberrant Activation of mTORC1 in NH B-Cell Lymphomas

#### Diffuse Large B Cell Lymphomas

Diffuse Large B Cell Lymphomas (DLBCLs) represent the most common and aggressive type of B lymphomas with 30–40% of the newly diagnosed NH B-cell lymphomas (WHO classification of tumors 2011) (5). DLBCLs are a genetically heterogeneous group of tumors, often associated with a deregulation of *BCL2* and/or *BCL6* and/or *MYC* genes, classified into specific subtypes that differ at the level of biology, histology and the clinical level. Compared to CHOP chemotherapy, R-CHOP immuno-chemotherapy has significantly improved the overall survival (OS) and the progression-free-survival (PFS) of patients with DLBCL, by 10–15% (84). However, 30–40% of patients suffering from DLBCL still experience therapeutic failure or relapse during R-CHOP therapy. Gene expression profiling has improved our understanding of DLBCL biology by classifying DLBCLs based on their cellular origin (Figure 1). Germinal center B-cell (GCB)-DLBCL shares a transcriptional profile similar to that of normal germinal center B cells, activated B-cell (ABC)-DLBCL expresses genes that are up regulated during the activation of normal B cells and Unclassified-DLBCL are distinct from the GCB or the ABC transcriptional profiles (85) (Figure 1). So far, there are limiting data demonstrating direct evidence of aberrant mTORC1 signaling in DLBCL biopsies. Immunohistochemistry (IHC) staining of phosphorylated-S6 protein (p-S6), one of the most sensitive targets of mTORC1, identified 62% of mTORC1 active DLBCL with a significant association with non-GCB-DLBCL (80%), while only 10% GCB-DLBCL stained positive for p-S6 (86). Consistent with the outcome of ABC-DLBCL, patients with mTORC1 active DLBCL who are treated with R-CHOP have an unfavorable outcome compared to those with mTORC1 inactive DLBCL (negative p-S6 staining) who show a strikingly long OS (60 months) (86). Mutations in mTOR (mostly missense mutations and truncating mutations) were reported in approximately 7% of human DLBCL, regardless of the DLBCL molecular subtypes (87). Consistently, knockout of mTOR impairs proliferation of ABC- and GCB-DLBCL lines (87). As for MCL, numerous indirect evidence of highly active mTORC1 signaling are demonstrated upstream of mTORC1 in DLBCL biopsies. Human primary DLBCL samples express heterogeneous levels of Rheb (mRNA and protein) and a high expression of Rheb is associated with aberrant activation of mTORC1 (88). Whether Rheb is differentially regulated in the specific molecular class of DLBCL remains unknown.

PTEN alterations were described in 10% of primary DLBCL and in 17% of DLBCL cell lines (89). PTEN loss is prevalent in GCB-DLBCL rather than ABC-DLBCL (90), a characteristic that was confirmed in other patient cohorts (91, 92). PTEN mutations are reported in 10.6% of DLBCL (12% of GCB-DLBCL and 9% of ABC-DLBCL) and correlate with the induction of genes involved in the regulation of Akt/mTOR signaling and metabolism (92). DLBCL harboring PTEN mutations do not significantly overlap with the cases exhibiting PTEN loss. Clinically, PTEN deletion or mutations are independent prognostic factors for poorer OS and PFS upon R-CHOP treatment in Akt activated DLBCL (92, 93). Less frequent, mutations in the *PIK3CA* gene or amplifications of PIK3CA are observed in 1.3–12% of DLBCL (93–96) and they are mutually exclusive with PTEN loss, which further define another PI3K/Akt/mTOR-regulated DLBCL subset (94). Importantly, PIK3CA mutations were undetected in a wide variety of human DLBCL cell lines (94), suggesting a heterogeneous mode of mTORC1 regulation that must be studied or at least confirmed in human primary samples rather than in human DLBCL cell lines.

Independently of Akt or MEK/ERK activation, mTORC1 signaling is constitutively activated in a fraction of GCB-DLBCL cells lines (and in Burkitt cell lines) (97) and in most of the ABC-DLBCL cell lines (98) (Figure 1). One hallmark of ABC-DLBCL is a constitutive activation of the NF-κB pathway (99) due to (i) mutations in NF-κB components CARD11 (100), A20 (101) or Myd88 (102) and/or (ii) chronic activation of the B-Cell Receptor (BCR) (as a result of mutations of in the BCR subunits CD79A/B) and downstream kinases (103, 104). In ABC-DLBCL, different scenarios regulate mTORC1 activation. Downstream of the BCR, the tyrosine kinase BTK (Bruton's Tyrosine Kinase) and the key NF-κB regulator IκB kinase, IKKβ, are required to integrate BCR and mTORC1 signaling (98). In a fraction of ABC-DLBCL cell lines, the serine/threonine kinase PIM2 (Proviral integration site for Moloney murine leukemia virus-2) is upregulated to sustain mTORC1 signaling (98). Overall, although regulated differently, mTORC1 signaling is activated in both ABC and GCB-DLBCL.

As previously discussed, whole genome arrays identified three other distinct DLBCL subsets: the oxidative phosphorylation (OxPhos) cluster, which is significantly enriched in genes involved in mitochondrial metabolism; the B cell receptor (BCR) signature, characterized by an up-regulation of cell-cycle regulatory genes and BCR components, later associated with glycolytic metabolism; and the Host Response (HR) cluster displaying components of the T-cell receptor and of molecules implicated in T/NK cell activation (38, 39). Consistent with the metabolic functions of mTORC1 involved in both OxPhos and glycolytic metabolism and with the mTORC1 activation observed in both ABC and GCB-DLBCL, it appears that cell-of-origin (COO) and BCR/OxPhos classifications are independent. However, it would be interesting to determine the extent to which mTOR signaling (mTORC1 and mTORC2) is induced in the BCR- and OxPhos-DLBCL, since unlike the BCR cluster, OxPhos-DLBCL do not express functional BCR. In addition, what specific aspects of their respective metabolism route contribute to mTORC1 activation in each cluster remains unknown. More recently, the loss of the tumor suppressor PP2A (serine/threonine protein phosphatatase 2A) defined another metabolic subset in B malignancies such as DLBCL, which repress the PPP and its corresponding anti-oxidant protection, thereby utilizing glucose-derived carbon though glycolysis (105).

#### Follicular Lymphomas

Follicular Lymphomas (FL) is the second most common type of NH B-cell lymphomas (30%) (5). It is an incurable malignancy with a median survival of 8–10 years. FL are low-grade germinal center B lymphomas accompanied by an immune cell infiltrate in the tumor microenvironment. FL are clinically characterized by an initial indolent phase often sensitive to Rituximab-based therapies, that might precede disease transformation into an aggressive form of lymphomas (DLBCL) in 30–40% of the cases (106), a risk that is not decreased by early initiation of the treatment. Neoplastic B cells quite systematically (90%) harbor the *t*(14;18) translocation, which recombines *BCL2* gene located at 18q21 with the immunoglobulin (Ig) H chain joining region at 14q32, leading to Bcl2 overexpression and apoptosis escape, a critical event in the development of FL. Other cytogenetic and epigenetic alterations enhancing cell growth and metabolism accompanied this translocation, such as in *MYC, TNFRSF14*, or *EZH2* (107). Similar to the mutations found in DLBCL, frequent mutations in signaling pathways including NF-κB are observed in FL. mTORC1 activity in FL is identified by a positive IHC staining of phosphorylated-S6K compared to tonsillar B cells issued from healthy donors (108). In FL, Syk activity is a critical regulator of mTORC1 signaling and independent of Akt activation (108). Activity of PKCζ also contributes to aberrant mTORC1 signaling in this disease (109, 110). Interestingly, somatic mutations in components of the mTORC1 complex were specifically enriched in FL (17%) (111), rarely in DLBCL (< 2%) (87, 111), while absent in other NH B-cell malignancies (111). These mutations occur in the gene RRAGC encoding a Ras-related GTP-binding protein (RagC). As a functional consequence, FL expressing RagC mutants increase Raptor binding, thus reinforcing mTORC1 signaling even in the absence of amino acids (111). While some pathways activating mTORC1 have been characterized in FL, the metabolism of those lymphomas remain unexplored. Such metabolic characterization of FL should help to understand the metabolic changes that might occur during FL transformation, a process which remains an open question.

#### Mantle Cell Lymphomas

Mantle Cell Lymphoma (MCL) is an aggressive and incurable lymphoma representing 5–10% of the NH B-cell lymphomas. Most patients with MCL are treated with Rituximab-based therapies such as R-CHOP. Unlike patients with other NH B-cell lymphomas, the outcome of patients with MCL is not improved following R-CHOP treatment (112), and the median overall survival (OS) still varies from 3–4 years. MCL are characterized by a *t*(11;14) (q13;q32) chromosomal translocation leading to the juxtaposition of the cyclin D1 gene on the chromosome 11 to the IgH chain enhancer region of the chromosome 14, thus leading to overexpression of the cyclin D1 mRNA and protein (113). Translation of the cyclin D1 mRNA is likely to be regulated by the mTORC1/4E-BP1 signaling axis (114). Constitutive activation of mTORC1 is observed in several human MCL cell lines *in vitro* (81). PTEN loss is reported in 65% of MCL patients displaying increased cyclin D1 translation (115) and it correlates with the constitutive activation of Akt/mTORC1 signaling in MCL patients and in human MCL cell lines (116). Furthermore, 68% of MCL patients and human MCL cell lines display an amplification of the PI3K catalytic subunit α (PI3K-p110α), encoded by the *PIK3CA* gene (117). An increased *PI3KCA* gene copy number is significantly associated with Akt phosphorylation and is mutually exclusive with PTEN loss (117) (Figure 1).

Identification of mTOR active B lymphomas seems to define a group of therapy-resistant tumors, thus linking mTOR activity to an unfavorable outcome for patients and providing a strong rationale for preclinical and clinical evaluation of mTOR-targeted therapies in refractory/relapsed NH B-cell malignancies.

## Targeting mTOR Signaling in NH B-Cell Lymphomas: From Preclinical Evidences to the Clinic

Rapamycin is a highly potent and selective inhibitor of mTORC1. It interacts and forms a complex with the 12kDa FK506-binding protein (FKBP12), which limits the access of substrates to the mTORC1 kinase active site. Rapamycin acts as an allosteric inhibitor of mTORC1 phosphorylation activities that are critical for proliferation (43, 118). Even if rapamycin itself is not able to inhibit mTORC2, long-term exposure to this drug might affect them TORC2 complex assembly and signaling in some cell types (45, 119) (Figure 4). Rapamycin was first approved in 2000 by the Food and Drug Administration (FDA) as an immunosuppressive agent for the prophylaxis of organ rejection in renal transplant patients. Due to the poor solubility and pharmacokinetics of rapamycin, a first generation of mTOR inhibitors, rapalogs (rapamycin analogs), were developed and evaluated in preclinical and in clinical studies. Phase 1 studies demonstrated that rapalogs induce manageable adverse effects such as asthenia, thrombocytopenia, microsites, hyperglycemia and hyperlipidemia. In 2007 and 2009, the FDA approved Temsirolimus (CCI-779, intravenously delivered) and Everolimus (RAD001, orally bioavailable), two water-soluble rapalogs, for the treatment of advanced renal cell carcinoma. In 2009, Temsirolimus was approved by the European Union for the treatment of refractory/relapsed MCL.

### Preclinical Evaluations of Rapalogs in the Treatment of DLBCL, FL, and MCL (*in vitro* and *in vivo* Studies)

Numerous studies demonstrated that the inhibition of mTORC1 with rapalogs induces cytostatic effects rather than cytotoxic responses in human DLBCL, FL, and MCL cell lines *in vitro* (81, 108, 115, 120–122). Rapalogs restrain cell proliferation of DLBCL regardless of the COO classification, and of the genetic alterations (98), a notion that is not sustained by the conclusions of a phase 2 study in patients with refractory/relapsed DLBCL treated with Everolimus (83). This raises a question about the sensitivity of cultured DLBCL cell lines to mTORC1 inhibition, as it might be the result of *in vitro* culture conditions that are promoting mTORC1 activity.

*In vivo* efficacy of Everolimus was evaluated in a genetically engineered mice model of *MYC*-induced lymphomagenesis, in the Eμ-*MYC* mice (123). Eμ-*MYC* mice develop spontaneous NH B lymphomas in 2–20 months after birth. They mimic a genetic feature of human Burkitt lymphomas (which harbor *MYC* translocation in most cases) and present morphologic characteristics close to human lymphoblastic lymphomas overexpressing *MYC*. Giving rise to immature B cells, Eμ- *MYC* lymphomas do not recapitulate features of other human NH B-cell lymphomas. Nevertheless, those lymphomas are unique at the molecular and metabolic levels, similar to human biopsies of NH B-cell lymphomas. Importantly, malignant Eμ-*MYC* cells can be easily transferred into WT syngeneic immuno-competent recipient mice in order to validate candidate cancer genes and to assess therapy efficacy (124). In transgenic mice, mTORC1 activity is required to initiate malignant transformation of B-lymphocytes (125). Daily administration of Everolimus (5 mg/kg, 6 days per week) significantly improves Eμ-*MYC*-bearing mice survival (upon adoptive transfer of Eμ-*MYC* cells) and the median OS widely varies from one Eμ-*MYC* lymphoma to another (125). The heterogeneity of Everolimus responses *in vivo* is likely related to the individual genetic alterations and the anti-apoptotic properties of each Eμ-*MYC* lymphoma. In agreement with *in vitro* studies, *in vivo* Everolimus activity is associated with a G1 cell cycle arrest but not with apoptosis. Importantly, while reducing the proportion of malignant B cells overexpressing *MYC*, Everolimus neither affects the number of mature B220 + CD19 + B cells of the bone marrow nor of the spleen in WT nor in Eμ-*MYC* mice (125). Moreover, despite its known function as an immunosuppressive agent, Everolimus treatment does not reduce the proportion of immune cells such as macrophages, activated T cells and NK cells both in WT and in Eμ-*MYC* mice, which demonstrates the specific effect of mTORC1 inhibition in tumor cells, while keeping the anti-cancer immune capacity of the mice intact (125).

Genetic mouse models recapitulating human genetic features of FL have been established (126, 127) but they are not used for preclinical studies because of their complexity and of their time to tumor development. Recently, patient-derived xenograft (PDX) models were established from different types of NH B-cell lymphomas. PDX models retain the similar genetic, histologic and clinical features of the original patient lymphomas and are compatible with preclinical studies on treatment-naïve or treatment-resistant tumors (128).

Collectively, the use of rapalogs as a single agent in the treatment of refractory/relapsed DLBCL, FL and MCL provided encouraging results in preclinical settings that supported further investigations in the clinic.

### Clinical Evaluation of Rapalogs Efficacy in the Treatment of Refractory/Relapsed NH B-Cell Lymphomas

In the view of the therapeutic emergency, refractory/relapsed MCL were the first B lymphoid neoplasm for which rapalogs (Temsirolimus and Everolimus) were evaluated as single-agent in clinical settings (78–82). Phase II trials demonstrated significant effects each rapalog alone, with 20–40% of overall response rates (ORR), cases of complete responses (CR) (3–10%) and manageable toxicities (Table 1). In a phase III trial, Temsirolimus was administrated at a dose of 175 mg weekly, over 3 weeks, followed by either 75 mg (175/75 mg) or 25 mg (175/25 mg) weekly (80). Each patient group was compared to patients treated with a single chemotherapeutic agent of the investigator's choice. Strikingly, ORR was 22 and 2% for patients treated with Temsirolimus 175/75 mg or investigator's choice, respectively (80). Despite a significant improvement of OS, PFS was significantly increased and therapeutic responses occurred in patients relapsing after several treatment lines with a Rituximab-containing regimen. This study ultimately led to the European Union approval (2009) of Temsriolimus as a single agent for the treatment of refractory/relapsed MCL.

Later, Temsirolimus demonstrated a substantial and significant anti-tumor activity in other refractory/relapsed NH B-cell lymphomas, such DLBCL and FL (81, 83) (Table 1). ORR was 28 and 54% for DLBCL and FL, respectively. CR (12 and 25%) and duration responses (7 and 12.5 months) were also higher in patients with FL than in those with DLBCL (83). These findings were also confirmed in a phase II study evaluating Everolimus in relapsed NH B-cell lymphomas (81).

Collectively, phase II trials indicated a more potent effect of rapalogs in inducing ORR and CR in low-grade lymphomas (FL) than in high-grade lymphomas (DLBCL or MCL) (Table 1). As FL arises from GCB cells, it might suggest that malignant B cell origin can influence the response to mTOR-targeted therapies. Further investigations would be needed to determine, for instance, the prognostic of ABC- and GCB-DLBCL treated with rapalogs in order to confirm that DLBCL- transformed from FL, i.e., mostly GCB-DLBCL, might be more sensitive than non-GCB-DLBCL to rapalogs (83). This will be helpful to advance progress on the use of mTOR-targeted therapy in the treatment of FL and DLBCL.

## Ongoing and Future Therapeutic Strategies Targeting mTORC1 in NH B-Cell Lymphomas

### Rational to Combine Rapalogs With Other Therapeutic Approaches

Although significant, the responsiveness of NH B-cell lymphomas to rapalogs remains modest in preclinical and in clinical settings. Consequently, rapalogs were clinically approved in only one type of NH B-cell lymphomas, the relapsed/refractory MCL for which standard therapies have failed. Explanations for this low efficacy can be collected from the molecular studies on tumor heterogeneity and they have to be taken into consideration with the intention of proposing novel therapeutic strategies combining rapalogs with relevant agents in order to improve patient's outcome. Knowing the role of mTORC1 and its downstream effectors in controlling processes required for cell survival, one expects to see an induction of cell death when rapalogs were used as a single agent. However, rapalogs only reduce cell proliferation and as soon as the treatment is discontinued, proliferation of tumor cells resumes (129). The cytostatic (rather than cytotoxic) effect of rapalogs has been described in many preclinical studies (*in vitro* and *in vivo*) using a variety of cancer cell lines, including NH B-cell lymphomas and it can be explained by two main mechanisms of cellular adaptations occurring upstream or downstream of mTORC1, thus raising two main potential therapeutic strategies to collapse tumor growth.

Firstly, the limited clinical success of rapalogs is likely related to mTORC1 upstream molecular events. Rapalogs release the mTORC1-regulated negative feedback loop on the upstream PI3K/Akt signaling (Figure 4). Sustained activity of mTORC2 also contributes to enhanced phosphorylation of Akt. Combined molecular events induce hyperphosphorylated Akt, which in turn does contribute to cell metabolism and survival in rapalogs-treated cells in many cancer cells lines, including human DLBCL cell lines (130). A second generation of mTOR inhibitors (ATP competitive inhibitors) have been developed to directly block the ATP-binding pocket of both mTORC1 and mTORC2, thus leading to inhibition of their catalytic activity. Since mTOR and PI3K share similarities in their sequence, ATP-competitive inhibitors block both kinases activities. As expected, such dual PI3K/mTOR inhibitors have higher efficacy than rapalogs in aggressive NH B-cell lymphomas *in vitro* and *in vivo* (131) but they have raised concerns of dose-limited toxicities that are thought to be linked to their low selectivity toward mTORC1 (132, 133).

Secondly, signaling events downstream of mTORC1 represent a second drawback of rapalogue therapy. Indeed, rapalogs only partially prevent the phosphorylation of certain mTORC1 substrates (Figure 4). For instance, they have a limited effect on 4E-BP1 phosphorylation while they abolish S6K phosphorylation. This was observed in several human cancer cell lines including human DLBCL cell lines, regardless of the ABC or GCB classification (122, 130, 134). Importantly, rapamycin-insensitive phosphorylation of 4E-BP1 is sufficient to stimulate cap-dependent translation (122, 134). Mechanistically, specific substrate sequences near the phosphorylation site of mTORC1, influence mTORC1 kinase activity and correlate with resistance to rapamycin-induced suppression of substrate phosphorylation (135).

**Figure 5:**
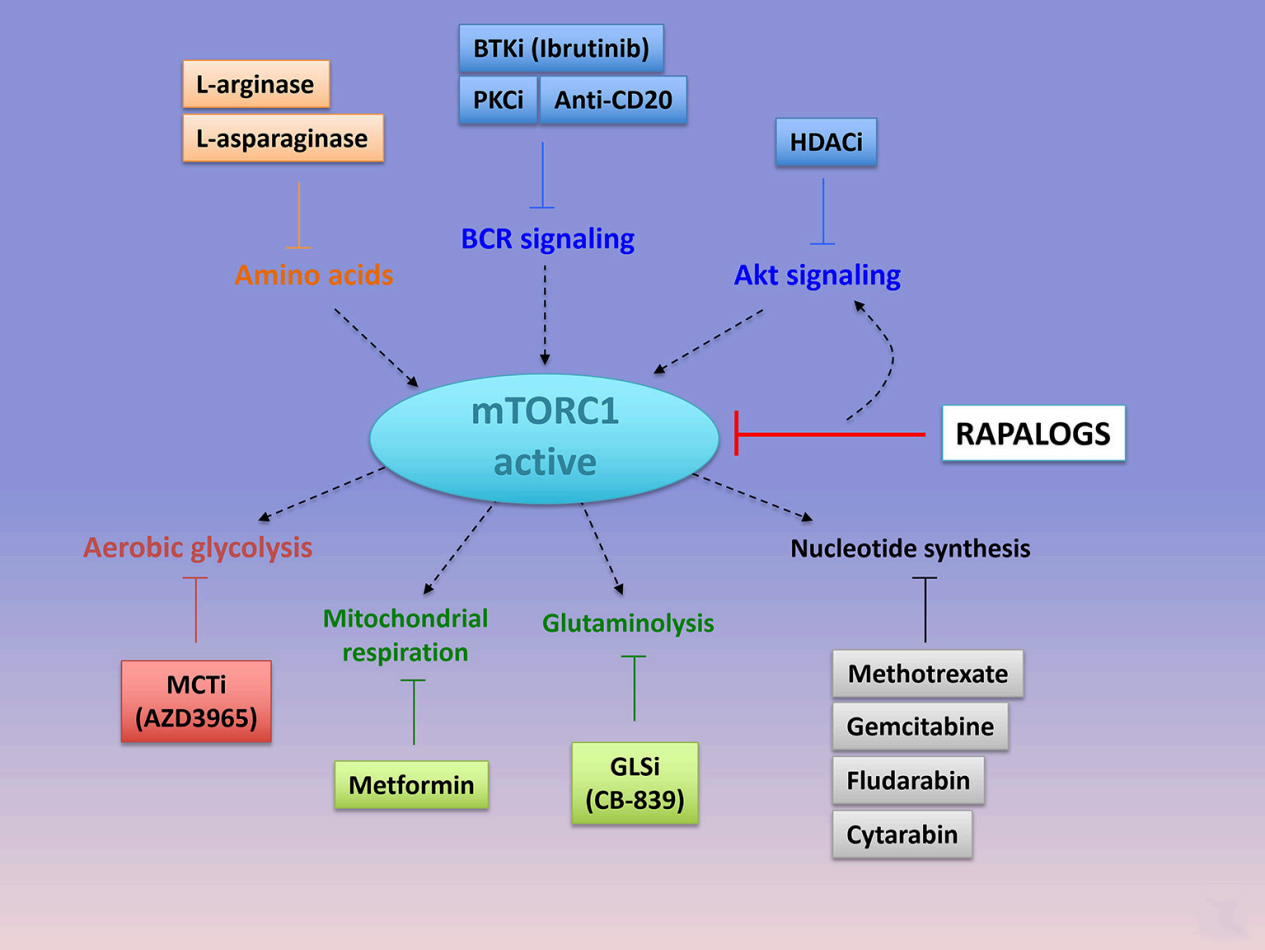
Emerging combinations with Rapalogs. Rapalogs only partially inhibit mTORC1 signaling. Already available drugs (approved or under clinical evaluation) can be combined with rapalogs in order to induce tumor cell killing. By inhibited S6K phosphorylation, rapalogs prevent the negative feedback loop on PI3K/Akt, resulting in PI3K/Akt activation. mTORC1 can be targeted with upstream kinases involved in BCR signaling (with anti-CD20 antibody or the PKC inhibitor AEB071, or the BTK inhibitor Ibrutinib) or in Akt signaling (with HDAC inhibitors, panobinostat or vorostinat). Hydrolysis of amino acids that are required for mTORC1 activation, such as arginine (with L-arginase), or glutamine (L-asparaginase), can further abolish mTORC1 signaling and induce cell death when combined with rapalogs. mTORC1-dependent regulation of metabolism is essential to induce an anabolic program promoting cell growth. Combined Rapalogs with inhibitor of glycolysis (MCT1 inhibitor, AZD3965), or mitochondrial complex I activity (Metformin) or gutaminolysis (GLS inhibitor, CB-839) or nucleotide synthesis (Methotrexate or Gemcitabine or Fludarabine or cytarabin), might improve patient's response. Importantly, the choice of the anti-metabolic strategy depends on the metabolic status of the tumor cells. PKC, Protein Kinase C; BTK, Bruton's Tyrosine Kinase; HDAC, histone deacetylase; MCT, Monocarboxylate Transporter.

Given the generally well-tolerated nature of rapalogs, combining the first generation of mTOR inhibitors with toxicities-manageable drugs targeting signaling or metabolic pathways upstream or downstream mTORC1, still represent valuable therapeutic approaches that might reposition rapalogs in the treatment of mTOR active tumors (Figure 5).

### Emerging Combinations With Rapalogs

We focus here on potentially relevant therapeutic strategies combining rapalogs with existing clinically approved agents or with new drugs under clinical evaluation (Tables 2, 3).

**Table 2:** Phase I/II and phase II studies (ongoing or published) evaluating the effect of rapalogs in combination with anti-CD20 or anti-CD20-based therapies.

**Disease**	**Therapeutic regimen**			**Trial phase**	**Nbe of patients in the cohort**	**ORR (%)**	**CR (%)**	**DR (months)**	**State of the study**	**Reference**
	**Rapalog**	**anti-CD20**	**chemotherapeutic agents**							
MCL	Temsirolimus	Rituximab	None	II	69	59	13	5.4 months (R-refractory) 10.9 months (R-sensitive)	published	(136)
DLBCL	Everolimus	Rituximab	none	II	24	38	12.5	8.1 months	published	(137)
MCL, CLL/SLL, DLBCL, Hodgkin's disease	Everolimus	Rituximab	none	II	49	-	-	-	active	NCT01665768
DLBCL	Temsirolimus	Rituximab	DHAP	II	88	-	-	-	active	NCT01653067
MCL, FL	Temsirolimus	Rituximab	Bendamustine	I/II	39	-	-	-	completed	NCT01078142

*MCL, Mantle Cell Lymphoma; DLBCL, Diffuse Large B Cell Lymphoma; FL, Follicular Lymphoma; CLL, Chronic Lymphocytic Leukemia; SLL, Small Lymphocytic Lymphoma; DHAP, Dexamethasone, High dose Ara-C and Platinol (cisplatin); ORR, overall response rate; CR, complete response; DR, duration response*.

**Table 3:** Anti-metabolic agents that can be combined with rapalogs to improve cytotoxic responses.

**Drug**	**Target**	**Status of the drug**	**Current indications**
**NUCLEOTIDE METABOLISM**			
Gemcitabine	Ribonucleotide reductase	Approved	Solid cancers (pancreatic, colon, lung) and NH B-cell lymphomas
Fludarabine	DNA synthesis	Approved	CLL, NH B-cell lymphomas
Cytarabin	DNA synthesis	Approved	AML, ALL, CLL, CML, and NH lymphomas
Metothrexate	Dihydroxyfolate reductase (DHFR)	Approved	Solid cancers (lung, breast, bladder, placenta), ALL
**AMINO ACID DEPLETION AND AMINO ACID METABOLISM**			
E-Coli-asparaginase Erwinase L-asparaginase, PEG-asparaginase	asparagine and glutamine	Approved	pediatric ALL Currently evaluated in adult leukemias, lymphomas and solid cancers
L-arginase	arginine	under clinical evaluation	Currently evaluated in pediatric ALL and AML, adult leukemia, lymphomas and solid cancers (kidney, liver)
CB-839	Glutaminase	under clinical evaluation	Current evaluation in ALL, AML, NH lymphomas and solid cancers (kidney, lung, skin, breast, colon)
**ENERGETIC METABOLISM**			
Metformin	ETC complex I	approved	Type 2 diabete Currently evaluated in childhood and adult leukemias, lymphomas and solid cancers
AZD3965	MCT1	under clinical evaluation	Currently evaluated in solid cancer, DLBCL, and Burkitt lymphomas

*NH B-cell lymphomas, Non-Hodgkin's B-cell lymphomas; CLL, Chronic Lymphocytic Leukemias; AML, Acute Myeloid Leukemia; ALL, Acute Lymphoblastic Leukemias; CML, Chronic Myeloid Leukemias*.

#### Combining Rapalogs With Anti-CD20-Based Therapies

Rapalogs were evaluated in combination with cytotoxic drugs inhibiting kinases upstream of mTORC1 to prevent rapalogs-induced reactivation of survival pathways and to promote tumor cell killing. For instance, MCL, DLBCL, and FL are currently treated with Rituximab-based chemotherapies such as R-CHOP. *In vitro*, Rituximab directly impact on cell viability of human CD20-expressing NH B-cell lymphoma cell lines by reducing (i) the expression of anti-apoptotic proteins Bcl-x, Bcl-2, XIAP, and Mcl-1 (138–140) and (ii) activity of kinases involved in BCR signaling (Lyn, Syk, Akt, Erk) (109, 141). *In vitro*, Everolimus sensitizes human GCB- and ABC-DLBCL cell lines to Rituximab-induced apoptosis by 10–20% (122, 130). Moreover, addition of Rituximab to Everolimus treatment prevents the reactivation of Akt at multi phosphorylation sites observed in Everolimus-treated cells. Consistently, combined Everolimus and Rituximab therapy is strikingly more efficient in reducing *in vivo* growth of human GCB-DLBCL cell lines xenografts in immunocompromised mice, than each treatment alone (130). This suggests that rapalogs can be used as an adjuvant to increase the cytotoxic effect of Rituximab, and thus provides a preclinical rationale for combining rapalogs and Rituximab clinically. Refractory/relapsed MCL treated with Rituximab as a single-agent demonstrated a ORR range from 27 to 37% (142–144). Phase II trials combining a rapalog and Rituximab in refractory/relapsed MCL (136) and DLBCL (137) are summarized in Table 2. At least for refractory/relapsed MCL, the responsiveness of this combination is higher (ORR 58 and CR 13%) than Rituximab alone or rapalog alone (Table 1), which warrants further investigations in randomized phase 3 trials, against Everolimus or Rituximab alone.

Rituximab is usually administered in combination with a chemotherapeutic regimen, mainly anthracyclines (Hydroxydaunorubicin), alkylating agents (cyclophosphamide or bendamustine) and/or anti-microtubule alkaloids (vincristine, also named oncovin). *In vitro*, a synergistically induction of apoptosis was observed in cultured human MCL cell lines in the presence of chemotherapeutic agents (doxorubicine or vincristine or paclitaxel) and Everolimus (121). A phase I study has evaluated the feasibility of Everolimus plus R-CHOP for new, untreated DLBCL. This therapeutic regimen did not show dose-limiting toxicities and demonstrated 96% of CR in a cohort of 24 evaluable patients (145). Ongoing phase I/II or phase II trials combining rapalogs with other anti-CD20-based therapies for the treatment of refractory/relapsed NH B-cell lymphomas are reported in Table 2.

#### Combining Rapalogs With Specific Inhibitors of Upstream Kinases: The Bruton's Tyrosine Kinase Inhibitor (Ibrutinib) or the PKC Inhibitor (AEB071)

The Bruton's tyrosine kinase (BTK) is required to transmit signals from the BCR to downstream kinases including PKC and thus integrates BCR and mTORC1 pathways in MCL, ABC-DLCL and CLL diseases. Consequently, combining mTOR and BTK inhibitions might collapse mTORC1 upstream signaling and mTORC1 activity. When combined with Everolimus or ATP-competitive inhibitors, the Bruton's tyrosine kinase (BTK) inhibitor (Ibrutinib) induces a synergistic effect on cell death in *in vitro* cultures of human ABC-DLBCL cell lines (146, 147). Although, this co-treatment holds a potential as promising therapeutic strategy, trials evaluating ibrutinib plus a rapalog are not reported so far. Interestingly, GCB-DLBCL are not sensitive to Ibrutinib and mTOR inhibitors.

We can also mention that a therapeutic strategy targeting PKC (with the PKC inhibitor AEB071) and mTORC1 (Everolimus), in CD79 mutant- or ABC-DLCBL is being investigated in a phase 1 trial (NCT01854606).

#### Combination of Rapalogs With the Histone Deacetylase (HDAC) Inhibitors

The FDA-approved HDAC inhibitors, panobinostat (LBH589) and vorostinat have been shown to prevent Akt signaling through mTORC2 in several cancer cell lines including DLBCL (148). As expected, DLBCL cell lines co-treated with rapamycin and the panobinostat (LBH589), display a synergistically inhibited phosphorylation of p70S6K and 4E-BP1 accompanied by an impaired cell survival (148). Temsirolimus or Everolimus also synergizes with vorostinat, in the treatment of MCL cell lines (121, 149). A phase I trial confirmed the effectiveness of this combination in a variety of refractory/relapse lymphomas, but with a cost of significant toxicity (150). A phase 2 study evaluating Everolimus and LBH589 in the treatment of refractory/relapsed DLBCL was aborted for toxicity reasons (NCT00978432).

#### Combining Rapalogs With Anti-metabolic Agents

The activation of both mTORC1 and mTORC2 pathways enhances cell metabolism (nucleotide, amino acid, and energetic metabolism), by regulating expression and/or activity of numerous metabolic enzymes to enable cell survival and growth. In turn, consequences of high metabolic activities allow persistent mTORC1 signaling. Since rapalogs are not able to fully abolish the function of certain mTORC1 substrates, there is a “leak” in the remaining metabolic fluxes, that can be directly targeted in order to further decrease the metabolic reprogramming regulated by mTOR and to prevent mTOR reactivation. Interestingly, targeting cell metabolism is of high clinical interest since many anti-metabolic drugs are already approved in the treatment of several types of cancers including B-cell malignancies or in the treatment of metabolic diseases and thus they can be easily evaluated in combination with rapalogs. Anti-metabolic drugs refer to any drug that might directly or indirectly target metabolic pathways.

##### Targeting nucleotide metabolism

Among the first effective chemotherapeutic agents identified are anti-metabolic drugs targeting nucleotides metabolism. By incorporating into DNA, RNA, or by inhibiting enzymes involved in nucleotide synthesis, this class of anti-metabolic agents slows purine and pyrimidine synthesis and DNA replication, thereby inducing cytotoxicity during the S phase of the cell cycle. Gemcitabine, a pyrimidine antagonist, inhibits DNA and RNA synthesis and causes cell cycle arrest. This drug is effective in the treatment of DLBCL when introduced in the therapeutic regimen R-GEMOX (Rituximab, Gemcitabine, Oxaliplatine). Fludarabine is a purine antagonist used in combination with Rituxmab and cyclophosphamide (R-FC) in the treatment of CLL. Cytarabin is a pyrimidine nucleoside analog routinely used in the treatment of AML. A phase 2 study combining Temsirolimus, Rituximab and high doses of cytarabin-based chemotherapy (DHAP), is currently ongoing in refractory/relapsed DLBCL [NCT01653067, (151)]. Not only purine and pyrimidine but also folic acid is necessary for the production of nucleotides. Methotrexate binds and inactivates an enzyme of folic acid metabolism, the dihydroxyfolate reductase (DHFR). This causes a decrease in DNA and RNA synthesis. Methotrexane is clinically used to prevent central nervous system relapse in patients with DLBCL. The combination of mTOR inhibitors and methotrexane were tested in acute lymphoblastic leukemia where it was synergistically effective (152). Administration of Temsirolimus and methotrexane leads to an increase OS and durable remission in immunocompetent mice xenografted with human AML patient samples (152).

##### Targeting energetic pathways

mTORC1 stimulates flux through different metabolic pathways including glycolysis, PPP, and OxPhos. Targeting mTORC1 and its downstream metabolic network might impair cellular energy production and tumor cell viability. Taking into consideration the existence of OxPhos tumor clusters (within a same tumor entity) and the OxPhos reliance of therapy-resistant tumors (41, 153), combining rapalogs with inhibitors of oxidative metabolism might enhance patient responses. The biguanide metformin is the best-characterized anti-metabolic agent that has found an application in the treatment of patients with type II diabetes (154). Metformin inhibits mitochondrial complex I activity, thereby decreasing cellular respiration, mitochondrial ATP production and increasing glucose uptake as a compensatory mechanism (155, 156). This last two decades, preclinical studies demonstrated that Metformin holds effective anti-proliferative activities in numerous human cancer cell lines, including NH B-cell lymphomas, thus repositioning Metformin in cancer prevention and treatment (157–161). Mechanistically, Metformin-induced energy depletion blocks mTORC1 activity in a AMPK-dependent or -independent manner, in physiological (162) and in pathological contexts (159, 163) In human DLBCL and Burkitt cell lines, Metformin inhibits mTORC1 and reduces cell proliferation in an AMPK dependent manner (164). Thus, combining Temsirolimus with Metformin shows a stronger *in vivo* growth inhibition than each treatment alone in B lymphoma xenografts (164). Other types of kinase inhibitors converging toward mTORC1 inhibition and biguanides synergistically impair tumor growth (165). In the clinic, indirect proof-of-concept of Metformin efficacy in DLBCL is demonstrated by a significant increase in PFS and CR of diabetic DLBCL patients on metformin compared to non-diabetic DLBCL patients (166). Phase 1 and phase 2 studies combining a rapalog and Metformin are currently ongoing in the treatment of several advanced solid cancers (NCT02048384; NCT01797523; NCT01529593), including lymphomas (NCT00659568).

Upon inhibition of glycolysis, mTORC1 sustains cell viability by reprogramming metabolism toward glutaminolysis and OxPhos metabolism. Inhibition of mTORC1 signaling decreases cellular respiration of glycolysis-independent cells. Co-targeting glycolysis and mTORC1 thus prevents metabolic escape and synergistically inhibits xenograft tumor progression (167). Although LDH-A represented a promising target for anti-cancer therapy (168, 169), none of the LDH-A inhibitors progressed in the clinic. Nevertheless, selective inhibition of the lactate transporter 1 (MCT1) with AZD3965 induces a negative feedback loop on the glycolytic rate and represents an alternative approach to target MCT4-defective glycolytic tumors (170). AZD3965 is currently under clinical evaluation in advanced solid cancers, in DLBCL and in Burkitt lymphomas (NCT01791595).

##### Targeting amino acids

Targeting extracellular sources of amino acids that are indirectly sensed by mTORC1 to regulate its activity represent another promising therapeutic option that might be used in combination with rapalogs to further prevent mTORC1 functions. So far, the key amino acids involved in mTORC1 regulation are arginase, leucine and glutamine. Glutamine uptake is required to uptake leucine and to promote leucine-dependent stimulation of mTORC1 at the lysosome. The L-asparaginase (*E*-*Coli*, Erwinase and other derivatives) hydrolyses extracellular asparagine and glutamine and prevents mTORC1 activation (63). L-asparaginase is highly effective in children undergoing induction therapy for acute lymphoblastic leukemias (ALL) and thus became a standard treatment for this childhood ALL. Severe adverse events associated with L-asparaginase reduced its clinical utilization in adults. Nevertheless, phase 2 studies evaluating L-asparaginase are currently ongoing for the treatment of multiple adult NH B-cell lymphomas and adult leukemias (NCT00018954 and NCT00002471). *In vitro* co-targeting of extracellular glutamine/asparagine and mTORC1 has, at least, additive effect, resulting in a stronger reduction of ALL cell lines viability (152). A phase 1 study is ongoing to evaluate the feasibility of combining Everolimus with chemotherapeutic agents including PEG(pegylated)-asparaginase in patients with ALL (NCT01523977). Similarly, L-arginase hydrolyses arginine and might further prevent mTORC1 activation in combination with rapalogs. It is worth mentioning that recombinant human arginase is under clinical evaluation in solid cancers (hepatocellular carcinomas, NCT00988195; liver cancers, NCT NCT00988195; melanoma and prostate adenocarcinomas, NCT02295101), in pediatric solid tumors, AML and ALL (NCT03455140) and in adult refractory/relapsed AML (NCT02899286).

Upon resistance to mTOR inhibition, glycolytic tumors upregulate the glutaminase (GLS) and switch to glutamine metabolism (171). The GLS inhibitor, CB-839 prevents the conversion of glutamine into glutamate and thus restricts carbon-derived glutamine sources required for *in vitro* TCA cycle anaplerosis and cell proliferation (19, 172–174). Inhibition of GLS activity overcomes resistance to mTOR inhibition (171) and provides a rational to target mTOR and GLS in metabolically flexible tumors. More generally, tumor cells overexpressing MYC are highly sensitive to GLS inhibition as they mainly rely on glutamine oxidation to replenish the TCA cycle, even in hypoxic conditions (19, 174). This suggests that patients with treatment-resistant *MYC*-translocated NH B-cell lymphomas might be susceptible to a combination of CB-839 and mTOR inhibitor. CB-839 is currently tested in a phase 1 trial in advanced and/or treatment refractory hematologic malignancies including NH lymphomas (NCT02071888) and is evaluating in combination with Everolimus in a phase 2 study in clear cell renal cell carcinomas (NCT 03163667).

It is important to note that therapeutic strategies combining rapalogs with anti-metabolic drugs hold potential as effective therapies only if we can better characterize the tumor metabolic state to address the best-adequate combination.

## Outlook and Conclusions

Targeting the metabolic control of tumor cells and more specifically of malignant B-cells hold potential as a promising anti-cancer strategy. As we discussed in this review, the first generation of mTORC1 inhibitors were effective *in vitro*, but the benefits provided to patients were often limited and quite disappointing. One of the reasons concerns the preclinical studies evaluating the impact of rapalogs on the survival of human transformed cells lines *in vitro*. All cell lines analyzed display increased mTORC1 signaling and show a reduction of cell proliferation upon mTORC1 inhibition. On the opposite, only a fraction of primary human NH B-cell lymphomas exhibit active mTORC1 signaling and are sensitive to rapalogs. It is likely that *in vitro* culture conditions, that provide nutrients in excess and stimulate the energetic and anabolic demands, select for rapid proliferating cells, glycolytic metabolism and chronic active mTORC1 signaling. However, *in vivo*, the intrinsic metabolic heterogeneity and the tumor microenvironment massively influence nutrient availability, which in turn modulates mTORC1 activation. Such biological factors should be taken into consideration when analyzing mTORC1 signaling and its inhibition in cancer. Thus, it is unlikely that *in vitro* culture of human transformed cell lines is relevant to the study of signaling pathways depending on nutrients to regulate cell metabolism and growth, as it might overestimate the effect induced by inhibition of these pathways. Moreover, the lack of robust pre-clinical models, that recapitulate the complex aspects of molecular heterogeneity of each human NH B-cell lymphomas, participates to the weakness of preclinical studies that evaluated *in vivo* efficacy of rapalogs in those malignances.

The absence of cytotoxic effect of rapalogs is also a main drawback. However, in rare cases, complete responses were obtained in patients with refractory/relapsed MCL, DLBCL, or FL, suggesting that some B lymphomas might be extremely sensitive to rapalogs probably because they display heavily active mTORC1 signaling. Identification of activating mutations in mTORC1 or of biomarkers of mTORC1 activity status will be helpful to predict the responsiveness of patients to rapalogs alone or in combination with other targeted therapies. Indeed, key information on rapalogs sensitivity can be collected from genetic investigations of tumor patients prior to mTOR-targeted therapies. Interestingly, tumors such as metastatic bladder cancers harboring the loss of function mutations in TSC1, are associated with Everolimus sensitivity in patients (175). In a phase 1 study, one patient with metastatic urothelial carcinoma refractory to current therapies presented a complete and durable (14 months) response to Everolimus and pazopanib treatment. Prior therapy, whole-exome sequencing of its extremely sensitive tumor revealed two activating mutations in mTOR (176). Such studies reinforce the idea that personalized medicine should be further considered in order to select patients who might respond to specific therapies. Importantly, 33 different mutations in mTOR components were recently reported in a variety of solid cancers (177). All these mutations confer hyperactivation of mTOR signaling. Although genetic alterations upstream of mTORC1 have been reported in different class of NH B-cell lymphomas, it might only represent a small proportion of mTOR active lymphomas. Obviously, the lack of a standardized approach to stratify patients with mTOR active tumors contributes in part to the limited clinical success of rapalogs. Since the diagnosis of NH B-cell lymphomas mainly relies on IHC staining of B cell specific markers in paraffin-embedded tumor sections (except for CLL that are diagnosed from blood samples of patients), determination of active or inactive mTORC1 signaling by IHC would be optimal. mTORC1 target phosphorylated-S6 was detected in 62% of newly diagnosed DLBCL (86). However, ORR is seen in only 30% of refractory/relapsed DLBCL (Table 1), raising two main questions far away from being solved. Firstly, is the level of mTORC1 activity different from the diagnosis to the relapsed after the first line therapies? Secondly, can we predict the response of NH B-cell lymphoma patients to rapalog by detecting mTOR targets in tumors? Different approaches (WB, IF, and IHC) to identify molecules related to mTOR activity such as mTOR, p-mTOR, and its targets, p-p70S6K, p-S6Rb, and p-4E-BP1 were compared in renal cell carcinoma biopsies using different approaches in order to find the best strategy to identify mTORC1 active tumors. It appears that only phosphorylated-S6K is a robust marker for detection of mTOR activity in tumor samples by at least two techniques including IHC, while the others molecules failed to be detected by IHC (178). Finally, the lack of robust and easily accessible biomarkers to evaluate the metabolic state of a given patient and, if possible, the heterogeneity of the metabolic status within the same tumor entity still represents a major block in the field. However, we can be confident that this missing gap will be documented in a near future.

## Author Contributions

All authors listed have made a substantial, direct and intellectual contribution to the work, and approved it for publication.

### Conflict of Interest Statement

The authors declare that the research was conducted in the absence of any commercial or financial relationships that could be construed as a potential conflict of interest.
